# Tumor necrosis factor induces rapid down-regulation of TXNIP in human T cells

**DOI:** 10.1038/s41598-019-53234-x

**Published:** 2019-11-13

**Authors:** Trine B. Levring, Martin Kongsbak-Wismann, Anna K. O. Rode, Fatima A. H. Al-Jaberi, Daniel V. Lopez, Özcan Met, Anders Woetmann, Charlotte M. Bonefeld, Niels Ødum, Carsten Geisler

**Affiliations:** 1 The LEO Foundation Skin Immunology Research Center, Department of Immunology and Microbiology, Faculty of Health and Medical Sciences, University of Copenhagen, Copenhagen, Denmark; 2 Center for Cancer Immune Therapy, Department of Oncology, Copenhagen University Hospital, Herlev, Denmark

**Keywords:** T cells, Signal transduction

## Abstract

In addition to antigen-driven signals, T cells need co-stimulatory signals for robust activation. Several receptors, including members of the tumor necrosis factor receptor superfamily (TNFRSF), can deliver co-stimulatory signals to T cells. Thioredoxin interacting protein (TXNIP) is an important inhibitor of glucose uptake and cell proliferation, but it is unknown how TXNIP is regulated in T cells. The aim of this study was to determine expression levels and regulation of TXNIP in human T cells. We found that naïve T cells express high levels of TXNIP and that treatment of blood samples with TNF results in rapid down-regulation of TXNIP in the T cells. TNF-induced TXNIP down-regulation correlated with increased glucose uptake. Furthermore, we found that density gradient centrifugation (DGC) induced down-regulation of TXNIP. We demonstrate that DGC induced TNF production that paralleled the TXNIP down-regulation. Treatment of blood with toll-like receptor (TLR) ligands induced TNF production and TXNIP down-regulation, suggesting that damage-associated molecular patterns (DAMPs), such as endogenous TLR ligands, released during DGC play a role in DGC-induced TXNIP down-regulation. Finally, we demonstrate that TNF-induced TXNIP down-regulation is dependent on caspase activity and is caused by caspase-mediated cleavage of TXNIP.

## Introduction

T cells circulate the body in a resting, naïve state until they recognize specific antigen/MHC complexes. For full activation and proliferation, naïve T cells need co-stimulatory signals in addition to antigen-driven signals. Many co-stimulatory receptors have been described on T cells^1^. Both tumor necrosis factor receptor 1 (TNFR1, CD120a) and TNFR2 (CD120b) can act as co-stimulatory receptors and accordingly, it has been described that TNF lowers the threshold for antigen-driven T cell activation and increases T cell proliferation and survival in both human and mouse T cells^2–8^. Although it has been suggested that TNF mediates its co-stimulatory effect by increasing the activation of the NFκB signaling pathway, other mechanisms might also be involved^9^. Resting T cells have low metabolic demands, relying mainly on oxidative phosphorylation. When activated, T cells proliferate and divide extensively, which greatly increases their metabolic demands. The increased metabolic requirements are met by up-regulation of glucose transporters accompanied by a significant increase in the glucose uptake as well as changes in the metabolism shifting from primarily oxidative phosphorylation to aerobic glycolysis^10–12^. High glucose uptake is essential in activated T cells as it provides the energy and building blocks required for proliferation and clonal expansion. In fact, limited glucose availability can prevent T cell proliferation and survival by stalling cells in G_1_ of the cell cycle^13,14^, making glucose uptake and utilization potential immunomodulatory targets^15^.

Thioredoxin interacting protein (TXNIP)^16^, also known as vitamin D up-regulated protein-1 (VDUP-1)^17^ or thioredoxin binding protein-2 (TBP-2)^18^, is a protein that plays a pivotal role in several cell processes including metabolism, growth, division, and apoptosis^19,20^. TXNIP can regulate the metabolism and division of cells by inhibiting their ability to take up glucose^21–24^. Additionally, TXNIP stabilizes the Cdk inhibitors p16 and p27, which inhibit cell division and keep the cells in G_1_^25,26^. These observations place TXNIP in the category of cell cycle inhibitors, a role which is supported by studies showing that TXNIP down-regulation is needed for cell division^27,28^, and that TXNIP functions as a tumor suppressor^19,29–31^. TXNIP expression is strongly and acutely regulated at the transcriptional level by several transcriptions factors, including MondoA, Mlx, FOXO1, and ChREBP^32^. Interestingly, pro-inflammatory factors down-regulate TXNIP in macrophages^33^, and it has been shown that TNF induces rapid down-regulation of TXNIP in the cell lines A549, HeLa, and NIH3T3^30,33,34^.

In previous studies, we have found that glutathione and thioredoxin are required for optimal T cell activation^35,36^. Combined with the observation that TXNIP binds and inhibits the reductase activity of thioredoxin^37^, this suggested that TXNIP plays important roles in regulation of T cell responses. However, only a few studies have touched on the function of TXNIP in T cells. One study indicated that TXNIP is expressed at high levels in naïve human T cells and that it is down-regulated in T cells activated through the T cell receptor^27^. In addition, studies of T cells infected with human T-lymphotropic virus type 1 (HTLV-1) indicated that TXNIP inhibited cell division, and that transformation from IL-2-dependent to IL-2-independent cell division was associated with a loss of TXNIP^25,38^. T cells from TXNIP knock-out mice divide more potently than T cells from wild-type mice, and TXNIP knock-out mice present with elevated lymphoid infiltration in the gut, indicating dysregulation of the T cells^39,40^. The aim of the this study was to determine the expression levels and regulation of TXNIP in human T cells.

Here, we provide first evidence that TNF induces rapid down-regulation of TXNIP and a concomitant increase in glucose uptake in naïve T cells. Importantly, TNF-induced TXNIP down-regulation is almost completely blocked by the caspase inhibitor Z-VAD-FMK indicating a mechanisms involving caspase-mediated cleavage of TXNIP. In addition, we show that purification of T cells by density gradient centrifugation (DGC) induces TXNIP down-regulation in the T cells probably due to the release of damage-associated molecular patterns (DAMPs) leading to secretion of TNF and other pro-inflammatory cytokines. Taken together, our data show that TXNIP is strongly expressed in naïve T cells but rapidly down-regulated by TNF, which suggests that TNF might act as a co-stimulatory molecule for T cells by inducing down-regulating of TXNIP.

## Results

### TNF induces TXNIP down-regulation and increased glucose up-take in T cells

**Figure 1:**
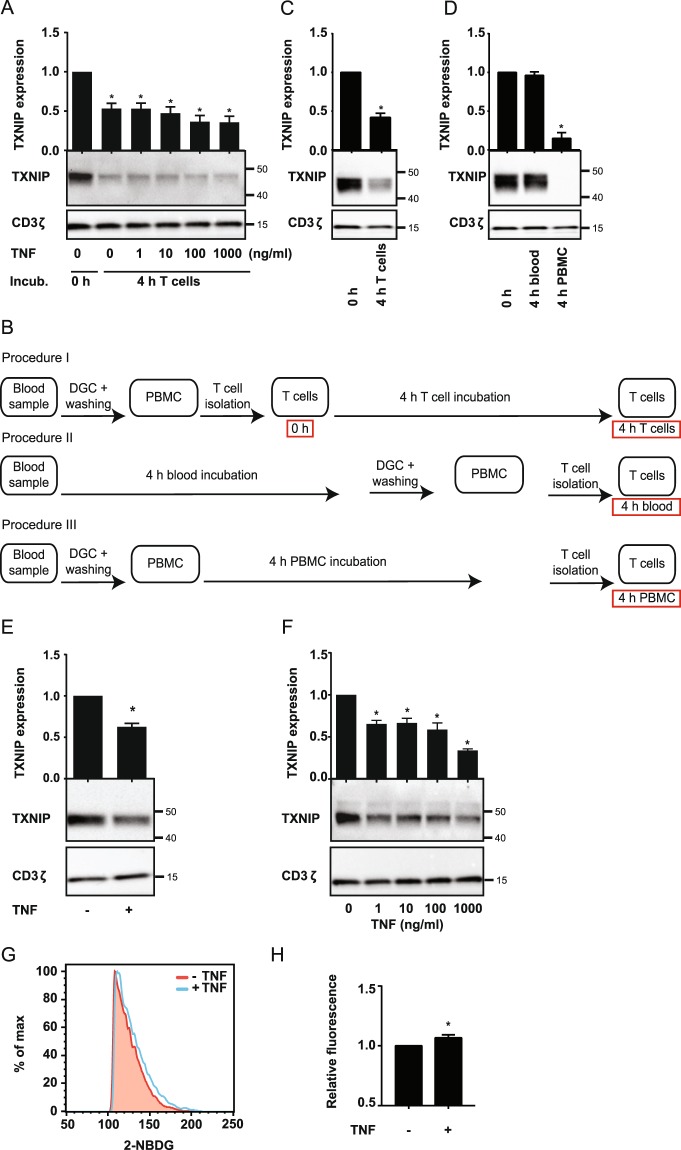
TNF induces TXNIP down-regulation and increased glucose up-take in T cells. (**A**) Representative Western blot (lower panel) and quantification (upper panel) of TXNIP with CD3ζ as loading control from T cells lysed immediately after isolation (0 h, Procedure I, **B**) and after 4 hours of incubation (4 h T cells, Procedure I, **B**) with TNF (0–1000 ng/ml) as indicated. (**B**) Overview of the different T cell isolation and incubation procedures used. The text in the red frames gives the nomenclature for the T cell lysates analyzed and shown in the figures. (**C**) Representative Western blot (lower panel) and quantification (upper panel) of TXNIP with CD3ζ as loading control from T cells incubated for 0 hours (0 h, Procedure I, **B**) and 4 hours (4 h T cells, Procedure I, **B**) before lysis. (**D**) Representative Western blot (lower panel) and quantification (upper panel) of TXNIP with CD3ζ as loading control from T cells immediately isolated and lysed (0 h, Procedure I, **B**), isolated and lysed after incubation of blood samples for 4 hours (4 h blood, Procedure II, **B**), and isolated and lysed after incubation of PBMC for 4 hours (4 h PBMC, Procedure III, **B**). (**E**) Representative Western blot (lower panel) and quantification (upper panel) of TXNIP with CD3ζ as loading control from T cells isolated from blood samples incubated for 4 hours (4 h blood, Procedure II, **B**) in the absence (−) or presence (+) of TNF (10 ng/ml). (**F**) Representative Western blot (lower panel) and quantification (upper panel) of TXNIP with CD3ζ as loading control from T cells isolated from blood samples incubated for 4 hours (4 h blood, Procedure II, **B**) with the indicated concentrations of TNF. (**F**) Representative FACS histograms and (**G**) relative mean fluorescence intensity of 2-NBDG^+^ T cells isolated from blood samples that were incubated with 2-NBDG in the absence (−) or presence (+) of TNF (10 ng/ml) for 4 h (4 h blood, Procedure II, **B**) (mean + SEM, n = 3). (**A–F**) Each Western blot is representative for Western blots obtained from at least 3 different biological experiments and the quantification shows the mean + SEM of the band densities of TXNIP from Western blots obtained from at least 3 different biological experiments. The positions of the relevant molecular weight markers and their molecular weight in kDa are given to the right of each Western blot.

Previous studies have found that TNF can induce TXNIP down-regulation in macrophages and cell lines^30,33,34^, and that TXNIP acts as an inhibitor of glucose uptake, tumor cell proliferation and cell cycle progression^26,30,31,41^. To determine whether TNF affects TXNIP expression in human T cells, we purified T cells from healthy donors, incubated them for 4 h in the absence or presence of TNF and subsequently determined their TXNIP expression levels by Western blot analyses. To our surprise, we found that T cells incubated for 4 h significantly down-regulated TXNIP compared to T cells incubated for 0 h independently of TNF (Fig. 1A). To verify this observation, we purified T cells and divided them in two equal samples. One sample was immediately lysed (0 h) whereas the other was incubated for 4 h (4 h T cells) without any other treatment (please see Fig. 1B for a detailed overview of the different isolation and incubation procedures used in the study). Again we found that TXNIP was significantly down-regulated in purified T cells incubated for 4 h (Fig. 1C). During isolation of purified T cells, we first isolate the peripheral blood mononuclear cells (PBMC) by density gradient centrifugation (DGC) and subsequently isolate the T cells from the PBMC. To determine at which point TXNIP down-regulation was initiated, we divided freshly drawn blood samples in three equal parts. From one part, the T cells were immediately isolated and lysed (0 h), the second part of the blood was incubated for 4 h before isolation and lysis of the T cells (4 h blood), and from the third part of the blood, the PBMC were isolated and then incubated for 4 h before the T cells were purified and lysed (4 h PBMC). Interestingly, we found that incubation of unprocessed blood for 4 h did not affect TXNIP levels (Fig. 1D, 0 h versus 4 h blood). However, incubation of the PBMC for 4 h before purification of the T cells resulted in an almost complete disappearance of TXNIP (Fig. 1D, 4 h PBMC). From these observations we concluded that purification of PBMC initiates TXNIP down-regulation in T cells and that the effect of TNF on TXNIP expression in T cells should be determined by incubating unprocessed blood samples with TNF. Interestingly, we found that treatment of unprocessed blood with TNF induced TXNIP down-regulation in the T cells (Fig. 1E). To further determine the effect of TNF, we treated blood samples with increasing concentrations of TNF for 4 h and subsequently purified the T cells and determined the TXNIP expression levels. We found that TXNIP expression was significantly down-regulated even at the lowest concentration of TNF tested (Fig. 1F). Previous studies have shown that TXNIP can regulate the metabolism and division of other cells than T cells by inhibiting their ability to take up glucose^21–24^. To determine whether TNF-mediated TXNIP down-regulation affected glucose up-take in T cells, we incubated blood samples with the fluorescent glucose analog 2-NBDG for 4 h in the absence or presence of TNF and subsequently determined the 2-NBDG uptake in the T cells by flow cytometry. We found that TNF significantly up-regulated the 2-NBDG uptake (Fig. 1G,H and Supplementary Fig. 2).

Taken together, these results demonstrated that TNF induces TXNIP down-regulation in T cells and that TNF-induced TXNIP down-regulation is associated with increased glucose up-take in T cells. In addition, the data indicated that DGC initiated TXNIP down-regulation by itself independently of TNF pre-treatment. As purification of PBMC by DGC is a key step in the majority of studies on human T cells, it is important to know whether and how this purification procedure affects the cells.

### DGC induces TXNIP down-regulation in T cells

**Figure 2:**
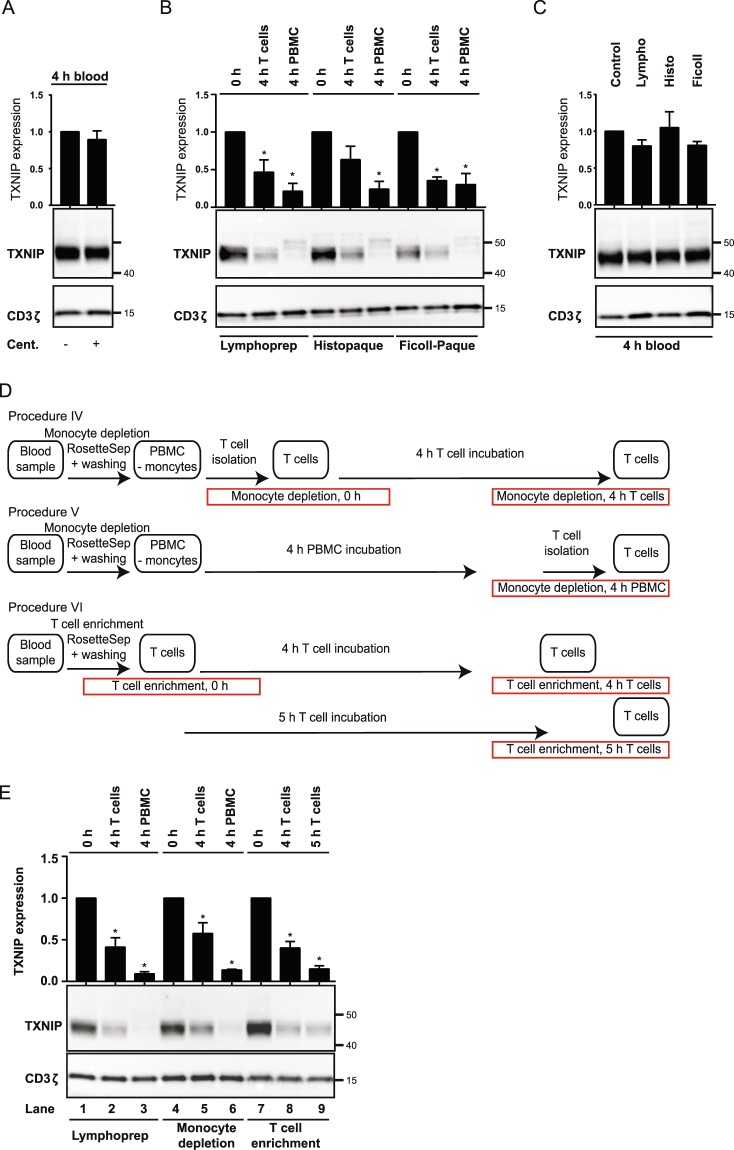
DGC induces TXNIP down-regulation in T cells. (**A**) Representative Western blot (lower panel) and quantification (upper panel) of TXNIP with CD3ζ as loading control from T cells isolated from blood samples incubated for 4 hours (4 h blood, Procedure II, Fig. 1B) after no treatment (−) or centrifugation (+). (**B**) Representative Western blot (lower panel) and quantification (upper panel) of TXNIP with CD3ζ as loading control from T cells immediately isolated and lysed (0 h, Procedure I, Fig. 1B), isolated and incubated for 4 hours before lysis (4 h T cells, Procedure I, Fig. 1B), and isolated and lysed after incubation of PBMC for 4 hours (4 h PBMC, Procedure III, Fig. 1B) after DGC on either Lymphoprep, Histopaque or Ficoll-Paque as indicated. (**C**) Representative Western blot (lower panel) and quantification (upper panel) of TXNIP with CD3ζ as loading control from T cells isolated from blood samples incubated for 4 hours (4 h blood, Procedure II, Fig. 1B) with no addition (Control) or with Lymphoprep, Histopaque or Ficoll-Paque as indicated. (**D**) Overview of different T cell isolation and incubation procedures used. The text in the red frames gives the procedure used and the nomenclature for the T cell lysates analyzed and shown in (**E)** lanes 4–9. (**E**) Representative Western blot (lower panel) and quantification (upper panel) of TXNIP with CD3ζ as loading control from T cells immediately isolated and lysed (lane 1: 0 h, Procedure I, Fig. 1B; lane 4: monocyte depletion, 0 h, Procedure IV, (**D)**; lane 7: T cell enrichment, 0 h, Procedure VI, **D**), isolated and incubated for 4 hours before lysis (lane 2: 4 h T cells, Procedure I, Fig. 1B; lane 5: monocyte depletion, 4 h T cells, Procedure IV, (**D)**; lane 8: T cell enrichment, 4 h T cells, Procedure VI, **D**), and isolated and lysed after incubation of PBMC for 4 hours (lane 3: 4 h PBMC, Procedure III, Fig. 1B; lane 6: monocyte depletion, 4 h PBMC, Procedure V, **D**) or T cells for 5 h (lane 9: T cell enrichment, 5 h T cells, Procedure VI, **D**). (**A–C**,**E**) Each Western blot is representative for Western blots obtained from at least 3 different biological experiments and the quantification shows the mean + SEM of the band densities of TXNIP from Western blots obtained from at least 3 different biological experiments. The positions of the relevant molecular weight markers and their molecular weight in kDa are given to the right of each Western blot.

To determine the mechanisms that initiated the rapid down-regulation of TXNIP during T cell isolation, we looked closer at the individual steps of the T cell isolation procedure. As in many other laboratories, we start with PBMC isolation that involves layering of heparinized blood diluted in PBS on a density medium followed by centrifugation. We routinely use the density medium Lymphoprep^TM^. We subsequently isolate the desired cells, here the T cells, from the PBMC by negative selection. In short, this involves coupling of magnetic beads to unwanted cells and then placing the cell suspension in a magnet. The unwanted cells are bound by the magnet enabling isolation of the desired, untouched T cells. From the result above, we knew that simple drawing and incubation of the blood did not induce TXNIP down-regulation (Fig. 1D, 0 h versus 4 h blood). Cells are exposed to strong centrifugal forces during isolation by DGC. To test whether the physical stress caused by the centrifugal forces induces TXNIP down-regulation, we divided blood samples in two parts. One part was centrifuged in the absence of density gradient medium prior to incubation for 4 h in order to mimic the centrifugal forces the cells are exposed to during the DGC isolation procedure, whereas the other part was simply incubated for 4 h without centrifugation. We found that centrifugation did not affect the level of TXNIP expression in the T cells (Fig. 2A), indicating that TXNIP down-regulation is not induced by the physical stress caused by ordinary centrifugation.

Since our observations indicated that TXNIP down-regulation was not caused merely by centrifugation of the cells, but by centrifugation of the cells on the density medium Lymphoprep^TM^, we next wanted to determine whether TXNIP down-regulation was specific for processing of the cells on Lymphoprep^TM^ or a common consequence of DGC. We consequently divided blood samples in three parts and centrifuged them on three different density media, namely Lymphoprep^TM^, Histopaque^®^–1077, and Ficoll-Paque^TM^, respectively. PBMC obtained from centrifugation on each of the density media were divided in three parts. From one part, T cells were immediately isolated and lysed (Fig. 2B, 0 h), and from the second part, T cells were immediately isolated and incubated at 37 °C for 4 h (Fig. 2B, 4 h T cells) before being lysed. The third part of PBMC was incubated for 4 h before isolation and lysis of the T cells (Fig. 2B, 4 h PBMC). The pattern of TXNIP expression was the same for all density media tested. TXNIP was clearly seen in T cells that were lysed directly after purification (Fig. 2B, 0 h), significantly down-regulated in T cells incubated for 4 h before lysis (Fig. 2B, 4 h T cells) and almost undetectable in T cells isolated from PBMC incubated for 4 h (Fig. 2B, 4 h PBMC). To exclude the possibility that TXNIP down-regulation was directly caused by substances in the density media, we divided blood samples into 4 parts and incubated them for 4 h with or without the different types of density media. None of the density media induced TXNIP down-regulation by simply incubating blood samples in their presence (Fig. 2C).

Taken together, neither centrifugation of blood nor physical contact of blood with the density media was sufficient to induce down-regulation of TXNIP in T cells. However, the combination of the two - centrifugation of blood *on* density gradient media – did induce TXNIP down-regulation. Since this effect was observed with 3 different kinds of density media, we concluded that TXNIP down-regulation in T cells is a general phenomenon when PBMC are isolated from human blood samples by DGC.

In an attempt to identify an alternative T cell purification procedure that does not induce TXNIP down-regulation, we tested the RosetteSep™ Human Monocyte Depletion Cocktail (monocyte depletion) and the RosetteSep™ Human T Cell Enrichment Cocktail (T cell enrichment) both from Stemcell. In the monocyte depletion procedure, whole blood is incubated with tetrameric antibody complexes recognizing CD36 on monocytes and glycophorin A on red blood cells. When subsequently centrifuged over a density medium such as Lymphoprep, the monocytes pellet along with the red blood cells and granulocytes resulting in a PBMC fraction depleted of monocytes. Likewise, in the T cell enrichment procedure, whole blood is incubated with a mixture of tetrameric antibody complexes recognizing non-T cells and glycophorin A. When subsequently centrifuged over a density medium, the non-T cell pellet along with the red blood cells and granulocytes resulting in a PBMC fraction depleted of non-T cells. PBMC obtained after the classic DGC centrifugation on Lymphoprep and cells obtained after using the monocyte depletion and T cell enrichment cocktails in combination with DGC were divided in three parts (please see Figs 1B and 2D for a detailed overview of the procedures used). From one part, T cells were immediately isolated and lysed (Fig. 2E, 0 h), and from the second part, T cells were immediately isolated and incubated at 37 °C for 4 (Fig. 2E, 4 h T cells) before being lysed. The third part of PBMC and cells obtained with the monocyte depletion cocktail was incubated for 4 h before isolation and lysis of the T cells (Fig. 2E, 4 h PBMC). The third part of the cells obtained with the T cell enrichment cocktail was incubated for 5 h before lysis of the T cells (Fig. 2E, 5 h T cells). The pattern of TXNIP expression was the same for all procedures tested. Thus, TXNIP was clearly seen in T cells lysed immediately after isolation in all three procedures, but was significantly down-regulated in T cells incubated for 4 and 5 h before being lysed. Likewise, monocyte depletion did not reduce the disappearance of TXNIP in T cells isolated after incubation of the PBMC for 4 h (Fig. 2E). Thus, we could not identify a T cell purification procedure that did not induce TXNIP down-regulation in the T cells, supporting that the role of TXNIP in T cells should be studied in unprocessed blood samples.

### DGC and TLR agonists induce TNF production and TXNIP down-regulation in T cells

**Figure 3:**
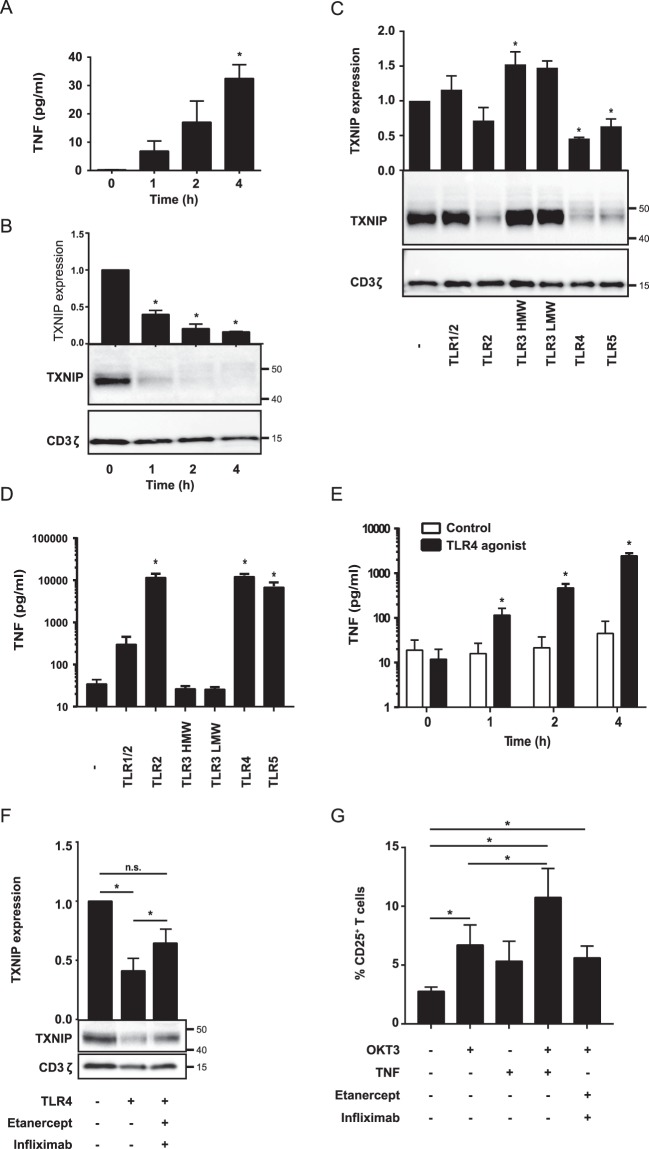
DGC and TLR agonists induce TNF production and TXNIP down-regulation in T cells. (**A**) TNF in the supernatant of PBMC incubated for 0 to 4 hours (mean + SEM, n = 3). (**B**) Representative Western blot (lower panel) and quantification (upper panel) of TXNIP with CD3ζ as loading control from T cells isolated from PBMC incubated for 0 to 4 hours. (**C**) Representative Western blot (lower panel) and quantification (upper panel) of TXNIP with CD3ζ as loading control from T cells isolated from untreated blood and blood treated with TLR1–5 ligands (TLR1/2, 500 ng/ml PamCSK4; TLR2, 1 × 10^8^ heat killed *Listeria monocytogenes*/ml; TLR3 HMW, 5 µg/ml Poly(I:C) high molecular weight; TLR3 LMW, 5 µg/ml Poly(I:C) low molecular weight; TLR4, 500 ng/ml *E. coli* K12 LPS; TLR5, 500 ng/ml *S. typhimurium* Flagellin) for 4 hours (4 h blood, Procedure II, Fig. 1B) as indicated. (**D**) TNF in the supernatant of blood incubated with the TLR1–5 ligands as above for 4 hours (4 h blood, Procedure II, Fig. 1B) as indicated. (**E**) TNF in the supernatant of blood incubated for 0 to 4 hours without (white columns) or with the TLR4 ligand (50 ng/ml) (black columns). (**F**) Representative Western blot (lower panel) and quantification (upper panel) of TXNIP with CD3ζ as loading control from T cells isolated from untreated blood and blood treated with the TLR4 ligand (50 ng/ml) in the absence or presence of the TNF inhibitors etanercept (10 µg/ml) and infliximab (10 µg/ml) for 4 hours (4 h blood, Procedure II, Fig. 1B) as indicated. (**G**) The frequency of CD25^+^ T cells in untreated blood, blood treated with OKT3 (100 ng/ml), TNF (10 ng/ml) and the TNF inhibitors etanercept (10 µg/ml) and infliximab (10 µg/ml) as indicated for 72 hours. The data show the mean + SEM obtained from four experiments with nine different donors. (**B,C,F**) Each Western blot is representative for Western blots obtained from at least 3 different biological experiments and the quantification shows the mean + SEM of the band densities of TXNIP from Western blots obtained from at least 3 different biological experiments. The positions of the relevant molecular weight markers and their molecular weight in kDa are given to the right of each Western blot.

As we had demonstrated that TNF induces TXNIP down-regulation, we decided to test whether TNF could be detected in the supernatant from PBMC isolated by DGC. Therefore, we incubated PBMC for 0 to 4 h after DGC and subsequently determined TNF in the supernatant and TXNIP expression levels in the T cells. TNF was clearly detectable in the supernatants after incubation for 1 h, and the TNF concentration increased with time correlating with a concomitant decrease in TXNIP expression (Fig. 3A,B).

It has been shown that DAMPs like endogenous TLR agonists are released from injured and dying cells^42–44^, and that monocytes and neutrophils can produce pro-inflammatory cytokines like TNF shortly after TLR activation^45,46^. Thus, if DGC causes cell injury or death, TLR on monocytes, neutrophils and probably other cell types in the blood could be activated by endogenous TLR agonists. TLR signaling might then via cytokine production cause TXNIP down-regulation in the T cells. In order to test this hypothesis, we incubated blood samples for 4 h in the absence or presence of TLR agonists 1–5 and subsequently measured TXNIP expression in the T cells and TNF in the supernatant. Indeed, we found that TLR2, 4 and 5 agonists induced TXNIP down-regulation (Fig. 3C) in parallel with TNF production (Fig. 3D). To determine whether TLR agonists could induce TNF as rapidly as TXNIP down-regulation was observed after DGC, we incubated blood samples with or without the TLR4 agonist for 0 to 4 h and subsequently measured TNF in the supernatant of the blood samples. Already at 1 h, the TLR4 agonist induced significant TNF production (Fig. 3E), further supporting the hypothesis that TLR-induced TNF production induces TXNIP down-regulation in T cells during DGC of human blood samples. To test this hypothesis further, we incubated blood samples with or without the TLR4 agonist for 4 h in the absence or presence of the TNF inhibitors etanercept and infliximab. We found that the TNF inhibitors partially blocked the TLR-induced TXNIP down-regulation (Fig. 3F). This observation confirmed that TLR-induced TXNIP down-regulation is partially mediated by TNF, but that other mechanisms/cytokines probably also are involved as a complete block in TXNIP down-regulation by use of the TNF inhibitors was not obtained.

Previous studies have shown that TNF can deliver co-stimulatory signals leading to enhanced CD25 expression in T cells^2,4,5^. To investigate whether TNF did deliver co-stimulatory signals in full blood, we incubated blood samples with the mitogenic anti-CD3 antibody OKT3 either alone or in combination with TNF or the TNF inhibitors etanercept and infliximab for 72 hours and subsequently determined the frequency of T cells that expressed CD25. We found that TNF in combination with OKT3 increased the frequency of CD25^+^ T cells approximately 2 fold compared to the frequency of CD25^+^ T cells in samples treated with OKT3 alone (Fig. 3G).

### TNF induces TXNIP down-regulation by a caspase-dependent mechanism

**Figure 4:**
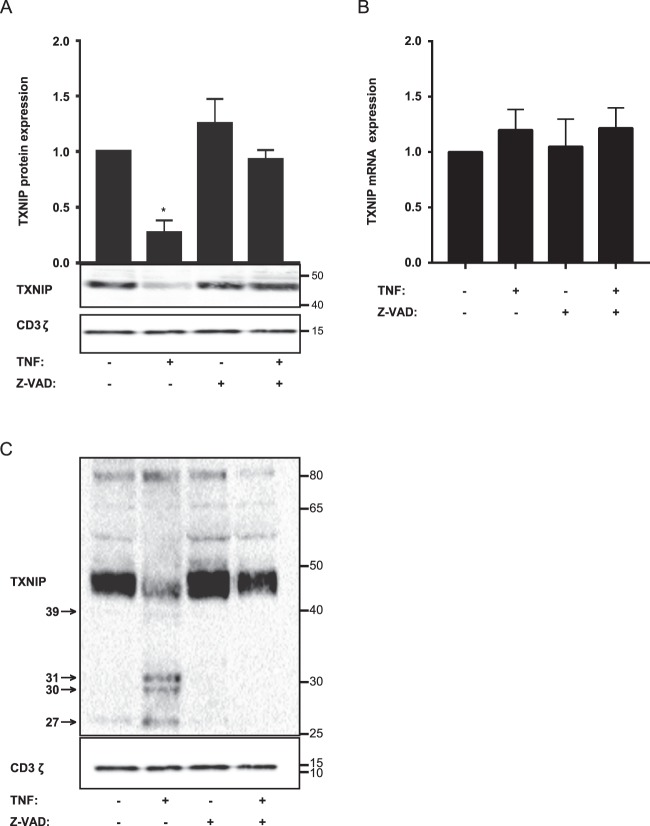
TNF induces TXNIP down-regulation by a caspase-dependent mechanism. (**A**) Representative Western blot (lower panel) and quantification (upper panel) of TXNIP with CD3ζ as loading control from T cells isolated from untreated blood and blood treated with TNF (10 ng/ml) and the caspase inhibitor Z-VAD-FMK (Z-VAD) (10 µg/ml) for 4 hours (4 h blood, Procedure II, Fig. 1B) as indicated. The Western blot is representative for Western blots obtained from 3 different biological experiments and the quantification shows the mean + SEM of the band densities of TXNIP from Western blots obtained from 3 different biological experiments. (**B**) Relative TXNIP mRNA expression in T cells isolated from untreated blood and blood treated with TNF (10 ng/ml) and the caspase inhibitor Z-VAD-FMK (Z-VAD) (10 µg/ml) for 4 hours (4 h blood, Procedure II, Fig. 1B) as indicated (mean + SEM, n = 3). (**C**) Overexposed Western blot of TXNIP with CD3ζ as loading control from T cells isolated from untreated blood and blood treated with TNF (10 ng/ml) and Z-VAD-FMK (Z-VAD) (10 µg/ml) for 4 hours (4 h blood, Procedure II, Fig. 1B) as indicated. The arrows indicate putative TXNIP fragments from caspase-mediated cleavage of TXNIP and their molecular weight in kDa. (**A,C**) The positions of the relevant molecular weight markers and their molecular weight in kDa are given to the right of each Western blot.

TNF-induced signaling comprises several signaling pathways in T cells including activation of caspases^8,47–49^. To identify the signaling pathways involved in the TNF-induced TXNIP down-regulation, we incubated blood samples with or without various cell signaling inhibitors in the absence or presence of TNF. Whereas inhibitors against p38, JNK, MEK, PI3K and IκB-α did not affect TNF-induced TXNIP down-regulation, the pan-caspase inhibitor Z-VAD-FMK completely abolished TNF-induced TXNIP down-regulation (Fig. 4A and Supplementary Fig. 3). In theory, TNF-induced caspase activation could either inhibit TXNIP expression by caspase-mediated cleavage of transcription factors important for TXNIP transcription^50–52^ or by caspase-mediated cleavage of TXNIP. Previous studies have indicated that TNF inhibits TXNIP transcription in cell lines^30,33,34^. However, by QPCR analyses we found that TNF and Z-VAD-FMK did not affect TXNIP transcription in T cells (Fig. 4B), indicating that transcription factors involved in TXNIP transcription were not affected by the activated caspases. In contrast, by overexposure of Western blots we found several low molecular bands in samples treated with TNF but not in the control samples or samples treated with TNF plus Z-VAD-FMK (Fig. 4C). These bands likely represent TXNIP fragments resulting from caspase-mediated cleavage of TXNIP. Accordingly, computational analyses using the programs Cascleave (http://sunflower.kuicr.kyoto-u.ac.jp/~sjn/Cascleave/index.html) and CasDB^53^ identified several caspase cleavage sites in TXNIP consistent with the size of the fragments observed in Fig. 4C.

## Discussion

In this study, we show that TNF induces rapid down-regulation of TXNIP in human T cells. The potential significance of TXNIP down-regulation in T cells is emphasized by the fact that previous studies have shown that TXNIP acts as a cell cycle inhibitor in other cell types, and that it needs to be down-regulated in order for cells to enter the cell cycle^19,25–27^. Several studies have described that TNF can lower the threshold for antigen-driven T cell activation and increases T cell proliferation and survival in both human and mouse T cells^2–8^, and it has been suggested that TNF mediates this effect by increasing the activation of the NFκB signaling pathway^9^. Our study suggests that in addition to its effect on the NFκB signaling pathway, TNF may acts as a co-stimulatory molecule by down-regulation of TXNIP. In various cell types, TXNIP has been shown to inhibit glucose uptake^21,27,54–56^, and cells stall in G_1_ of the cell cycle if not enough glucose is available^57,58^. T cell activation is highly dependent upon glucose uptake. Increased glucose uptake is detectable in T cells within 1 hour of activation^59^, and limited glucose availability can prevent T cell activation and proliferation^14^. We found that TNF, in parallel with TXNIP down-regulation, induced increased glucose uptake in otherwise untreated T cells. This suggested that one of the mechanisms behind the co-stimulatory effects of TNF might be an increased glucose uptake in the T cells due to TNF-induced TXNIP down-regulation. Most probably several other mechanisms are involved in the co-stimulatory effect of TNF as for instance the enhanced expression of CD25 as described previously^2,4,5^ and by us in the present study.

We further found that DGC induces TXNIP down-regulation. The effect was observed with 3 different kinds of density media, indicating that TXNIP down-regulation in T cells is a phenomenon induced by DGC in general. Other studies have indicated that DGC can affect cells as measured by up-regulation of adhesion molecules on T cells^60^, internalization of chemokine receptors on monocytes and lymphocytes^61^, down-regulation of L-selectin on lymphocytes^62^, and increased expression of chemokine receptors on NK cells^63^. However, we are not aware of previous studies that found such a rapid and distinct down-regulation of an intracellular protein as we present here. It could be speculated that the density media might be polluted with endotoxins or other substances that would affect the cells during the purification procedure. However, we could exclude that the density media contained substances that directly induced TXNIP down-regulation as incubation of blood with the density media did not by itself induce TXNIP down-regulation. Neither did ordinary centrifugation of the blood induce TXNIP down-regulation. Only the combination of centrifugation on the density media induced TXNIP down-regulation. Further, we found that treatment of blood samples with TLR2, 4, and 5 agonists rapidly induced TNF production concomitantly with TXNIP down-regulation. This suggested that DGC might induce cell injury that could result in the release of DAMPs such as endogenous TLR ligands leading to TLR signaling and induction of TXNIP down-regulation. It has been reported that monocytes can produce pro-inflammatory cytokines like TNF shortly after TLR triggering^45^, and we found that PBMC produced significant amounts of TNF shortly after DGC. This is in accordance with another study, which found that PBMC isolated by DGC spontaneously produced TNF, IL-1β and IL-6^64^. We could only partially inhibit TLR-mediated TXNIP down-regulation by blocking TNF signaling in blood samples with the TNF inhibitors infliximab and etanercept, suggesting that other cytokines in addition to TNF can induce TXNIP down-regulation. This is in accordance with a study by Kanari *et al*., which found that both TNF and IL-1β induced TXNIP down-regulation in the mouse fibroblast cell line NIH3T3^33^. It is furthermore supported by our observation that PBMC isolation and incubation induced significantly more TXNIP down-regulation than treatment of the blood with TNF did. Furthermore, our study indicated that TXNIP down-regulation is already initiated in the T cells during the DGC as we could not prevent TXNIP down-regulation by use of the monocyte depletion and T cell enrichment cocktails. Although our data strongly point to DGC-induced cytokine production as the main initiator of TXNIP down-regulation in the purified T cells, we cannot formally exclude the possibility that by isolating the T cells, we remove factors that normally prevent TXNIP down-regulation in T cells. However, we can conclude that TNF induces rapid down-regulation of TXNIP in T cells. It is generally assumed that T cells isolated by DGC are not affected by the procedure and are equivalent to T cells found in the circulation. However, our data indicate that DGC causes TXNIP down-regulation that probably leaves the T cells primed and easier to activate subsequently. This is supported by the observation that T cells from PBMC isolated by DGC can be activated by lower levels of CMV antigen than T cells from whole blood^65^. The fact that purification of T cells greatly affects a central metabolic regulator as TXNIP might be important to take into consideration when studying T cell metabolism and activation *in vitro*.

In line with our observations, previous studies have found that TNF can induce TXNIP down-regulation in macrophages and the A549, HeLa, and NIH3T3 cell lines^30,33,34^. However, the mechanisms behind TXNIP down-regulation described in these studies differ from our results. Whereas we found that TNF did not affect *TXNIP* transcription but caused TXNIP down-regulation by caspase-mediated cleavage, they found that TNF inhibited *TXNIP* transcription and suggested that this might be the major mechanism in addition to increased TXNIP degradation. One explanation for these opposing observations could be that our study was made in primary human T cells and not in immortalized cell lines. It is known that thioredoxin binds to and protects TXNIP from degradation^28,66^. In contrast to immortalized cell lines that express high levels of thioredoxin, naïve human T cells do not express thioredoxin at detectable levels^36^ meaning that TXNIP is not protected by thioredoxin in naïve T cells.

In conclusion, in this study we show that TXNIP is highly expressed in naïve human T cells and that TNF induces rapid down-regulation of TXNIP in the cells. Furthermore, we show that purification of PBMC by DGC induces TNF production and TXNIP down-regulation in the T cells. We propose that down-regulation of TXNIP represents the release of one of several brakes on cell cycle progression and that TXNIP down-regulation might be one of the mechanisms behind the co-stimulatory effect of TNF in T cells.

## Methods

### Chemicals

TNF (210-TA) was from R&D Systems (Abingdon, United Kingdom), TLR1–5 agonists (tlrl-kit1hw), SB203580 (tlrl-sb20), SP600125 (tlrl-sp60), PD98059 (tlrl-pd98), LY294002 (tlrl-ly29), Bay11–7082 (tlrl-b82) and Z-VAD-FMK (tlrl-vad) were from InvivoGen (Toulouse, France). Infliximab and etanercept were obtained from Region Hovedstadens Apotek, Herlev, Denmark. The mitogenic anti-CD3 monoclonal antibody OKT3 (16-0037-85) was from ThermoFisher Scientific, USA.

### Cells and cell purification

Blood samples were obtained from healthy volunteers after obtaining informed, written consent in accordance with the Declarations of Helsinki principles for research involving human objects. The study was approved by The Committees of Biomedical Research Ethics for the Capital Region in Denmark (H-16033682). Heparin (585679, LEO Pharma A/S, Ballerup, Denmark) was added to the blood samples. For some experiments, the blood was incubated for 4 h at 37 °C in 5% CO_2_ before proceeding to DGC. For DGC, blood was diluted 1:1 in PBS + 0.15% BSA before being layered over density medium in SepMate^TM^–50 tubes (15460, Stemcell). The tubes contained 15 ml density medium and up to 25 ml diluted blood was layered over. Unless otherwise specified, Lymphoprep^TM^ (1115758, AXIS-SHIELD) was used, and the tubes centrifuged for 10 min at 1200 g, 24 °C with the brake on as specified by the manufacturers’ instructions. Where indicated, Histopaque^®^–1077 (10771, Sigma-Aldrich) or Ficoll-Paque^TM^ (GE17-5442-02, Sigma-Aldrich) was used and centrifuged for 30 min at 400 g, 24 °C with the brake off as specified by the manufacturers’ instructions. Following DGC, the top layer containing the PBMC was poured off, diluted 1:1 in PBS + 0.15% BSA, and centrifuged for 10 min at 350 g, 24 °C. The cell pellet was subsequently resuspended in PBS + 0.15% BSA and centrifuged for 10 min at 300 g, 24 °C. The PBMC were subsequently resuspended in PBS + 0.5% BSA + 1 mM EDTA at a concentration of 5 × 10^7^ cells/ml. The T cells were either isolated directly from this cell suspension or, where indicated, the PBMC were incubated up to 4 h at 37 °C in 5% CO_2_, before proceeding with T cell isolation. We used negative selection to purify untouched T cells. 50 µl EasySep™ Human T Cell Enrichment Kit (19051, Stemcell) was added per ml PBMC and incubated for 10 min, followed by addition of 50 µl/ml magnetic particles and 10 min incubation. The total volume was then brought to 5 ml by addition of PBS + 0.5% BSA + 1 mM EDTA and the tubes placed in magnets (18001, Stemcell) for 10 min. After incubation in the magnets, the liquid was poured off into a second tube. The remaining beads and cells from the first tube were resuspended in 5 ml PBS + 0.5% BSA + 1 mM EDTA and placed in magnets for another 10 min before being poured off into the second tube. The second tube, now containing 10 ml, was then placed in the magnet for 10 min, before finally pouring off the liquid containing the desired cells. All the incubation steps were carried out at room temperature. We obtained a purity of over 90% T cells by using this method. As alternative T cell isolation procedures, we tested the RosetteSep™ Human T Cell Enrichment Cocktail (15061, Stemcell) and the RosetteSep™ Human Monocyte Depletion Cocktail (15668, Stemcell) according to the manufacturer’s instructions. When using the monocyte depletion cocktail, T cells were subsequently enriched using the EasySep™ Human T Cell Enrichment Kit as described above. For all isolation procedures used, the purified cells were resuspended in X-VIVO 15 medium (1041, Lonza, Verviers, Belgium) and placed in 24-well plates (142475, Thermo Fisher Scientific, Roskilde, Denmark). Cells were incubated at 37 °C in 5% CO_2_. In the experiments with TLR agonists, the final concentrations used were 500 ng/ml PamCSK4 (TLR1/2 agonist), 1 × 10^8^ heat killed *Listeria monocytogenes*/ml (TLR2 agonist), 5 µg/ml Poly(I:C) high molecular weight (TLR3 agonist HMW), 5 µg/ml Poly(I:C) low molecular weight (TLR3 agonist LMW), 500 ng/ml *E. coli* K12 LPS (TLR4 agonist), and 500 ng/ml *S. typhimurium* Flagellin (TLR5 agonist).

### Western blot

Western blot analysis was carried out as previously described^35,36^. In short, cells were lysed in 1% Triton X-100 lysis buffer with Protease/Phosphatase Inhibitor Cocktail (5872 S, Cell Signaling). Before lysis the cells were counted and adjusted so that 1 × 10^6^ cells were loaded per lane. The samples were run on 10% polyacrylamide gels. The proteins were transferred to Amersham Hybond ECL nitrocellulose sheets (RPN2020D, GE Healthcare, Brondby, Denmark). The sheets were cut according to the molecular weight of the specific protein before probing with specific antibodies and finally visualized using ECL technology (RPN2232, GE Healthcare). For band density quantification ECL exposed sheets were analyzed in a ChemiDoc MP Imaging System from Bio-Rad. Primary antibodies used included mouse anti-TXNIP antibody (1:1000) (K0205-3, MBL/Medical & Biological Laboratories) and mouse anti-CD3ζ (1:2000) (6B10.2, Santa Cruz Biotechnology, Santa Cruz, CA). Secondary antibodies used were HRP-conjugated polyclonal rabbit anti-mouse Ig (1:2000) (P0260, DAKO, Glostrup, Denmark). In our hands, CD3ζ is the best control for number of T cells loaded, as CD3ζ expression is not significantly affected by T cell activation or differentiation^67–69^ or the experimental procedures used in the present paper (Supplementary Fig. 1). The Western blots shown in the figures are representative for Western blots obtained from at least 3 different biological experiments and likewise the quantification data of the band densities of TXNIP were obtained from at least 3 different biological experiments.

### Flow cytometry and glucose uptake analyses

Antibodies for flow cytometric analyses were CD3-APC (300412, Biolegend, San Diego, CA, USA), CD4-PE (555347, BD Biosciences, Franklin Lakes, NJ, USA), CD8-FITC (555366, BD Biosciences), and CD25-PE-Cy7 (302612, Biolegend). Viability dye eFluorTM 780 (65-0865-14, eBiosciences, San Diego, CA, USA) was used to exclude dead cells in the analysis. For glucose uptake experiments the fluorescent glucose analog 2-NBDG (2-(N-(7-nitrobenz-2-oxa-1,3-diazol-4-yl)amino)-2-deoxyglucose (N13195, ThermoFisher Scientific Inc., IL, USA) was used. Freshly drawn blood was diluted 1:1 in X-VIVO 15 medium and left unstimulated or stimulated with either TNF (10 ng/ml) or OKT3 (1000 ng/ml) and LPS (50 ng/ml). The cells were incubated either in the presence or absence of 10 μM 2-NBDG. After 4 hours, the PBMC were isolated using Lymphoprep and subsequently stained for flow cytometric analysis. In short, the cells were washed in PBS containing 0.2% sodium azid and 2% fetal bovine serum (FACS/PBS) and hereafter stained with fluorophore-conjugated antibodies for 30 minutes at 4 ^o^C protected from light. Following staining, the cells were washed twice in FACS/PBS to remove unbound antibodies. As gating strategy, lymphocytes were first gated in the forward-scatter (FSC) side-scatter (SSC) dot plot. Subsequently singlet cells were isolated and next dead cells were gated out based on viability staining. Lastly, CD3^+^ T cells were gated and analyzed for 2-NBDG uptake with the positive gate set in relation to cells incubated in the absence of 2-NBDG (Supplementary Fig. 2). For CD25-upregulation experiment, freshly drawn blood was diluted 1:1 in X-VIVO 15 medium and left unstimulated or stimulated with either TNF (10 ng/ml), OKT3 (100 ng/ml), a combination of TNF and OKT3 or with OKT3 in combination with eternacept (10 μg/ml) and infliximab (10 μg/ml) 72 hours. The same staining procedures and gating strategy were applied as in the glucose up-take experiments (Supplementary Fig. 2), except that T cells were identified on their CD4 or CD8 expression as CD3 staining was blocked by the stimulatory OKT3 antibody. All samples were analyzed on a BD LSR II at the Core Facility for Flow Cytometry, Faculty of Health and Medical Sciences, University of Copenhagen. Data was analyzed using the FlowJo software (Treestar, Ashland, OR, USA).

### TNF ELISA

For detection of TNF, the Human TNF-α High Sensitivity ELISA kit (BMS223HS, eBioscience, AH diagnostics, Copenhagen, Denmark) was used and carried out according to the manufacturer’s protocol. In short, pre-coated plates were washed twice before addition of samples and standard in duplicates. Biotin-conjugate was then added to all wells and the plate incubated for 2 h at room temperature on a microplate shaker set to ~400 rpm. After washing, Streptavidin-HRP was added to all wells and incubated for 1 h at room temperature on a microplate shaker. The plate was washed again before addition of Amplification Solution I and 15 min incubation at room temperature on a microplate shaker. After further washing, Amplification Solution II was added and the plate incubated for 30 min at room temperature on a microplate shaker. After a final wash, TMB Substrate Solution was added and the plate incubated for about 10–20 min in the dark. Stop Solution was added to stop the reaction and the plate was read immediately after on a Multiscan™ FC Microplate Photometer (type 357, Thermo Scientific). Absorbance was determined at 450 nm as the primary wavelength and the absorbance at 620 nm was used as the reference wavelength.

### QPCR

For extraction of RNA, cells were lysed in TriReagent (T9424, Sigma Aldrich) and vortexed thoroughly. 1-bromo-3-chloropropane (BCP) (B9673, Sigma Aldrich) was added to separate the sample into an aqueous and an organic phase. The RNA was precipitated from the aqueous phase using isopropanol, washed with ethanol and dissolved in RNase free water. Synthesis of complementary DNA (cDNA) was performed using the High Capacity RNA-to-cDNA Kit from Applied Biosystems^TM^ (4387406, ThermoFisher Scientific). For qPCR, 12.5 ng cDNA was mixed with TaqMan® Universal Master Mix II, with UNG (4440038, Thermo Fisher Scientific), target primer and the 18 S control primer. The samples were run on a Stratagene Mx3000P^TM^ real-time PCR machine (Agilent Technologies). The thermal profile was set to 2 min at 50 °C, a 10 min hot start at 95 °C, followed by cycles of 15 sec at 95 °C and 1 min at 60 °C. Signal intensity was measured at the end of the 60 °C step, and the threshold cycle (Ct) values were related to the control primer eukaryotic 18 S rRNA, which was included in each sample. The TXNIP (Hs01006897_g1) and eukaryotic 18 S rRNA (4319413E) primers from Thermo Fisher Scientific were used.

### Statistical analysis

Data are shown as mean + SEM. Significance levels were calculated in GrahPad Prism using paired t test. *Indicates p ≤ 0.05; n.s. indicates not significant.

## Supplementary information

Supplementary info
