# Supplementary material for: Tumor necrosis factor induces rapid down-regulation of TXNIP in human T cells
Source: Sci Rep. 2019 Nov 13;9:16725. doi: 10.1038/s41598-019-53234-x (PMC6853882; doi:10.1038/s41598-019-53234-x)
Supplement: Supplementary file 1 — Supplementary info [file 41598_2019_53234_MOESM1_ESM.pdf]

Tumor necrosis factor induces rapid down-regulation of TXNIP in human T cells

Trine B. Levring[1], Martin Kongsbak-Wismann[1], Anna K. O. Rode[1], Fatima A. H. Al-Jaberi[1], Daniel V. Lopez[1], Özcan Met[1, 2], Anders Woetmann[1], Charlotte M. Bonefeld[1], Niels Ødum[1] and Carsten Geisler[1*]

Supplementary Info

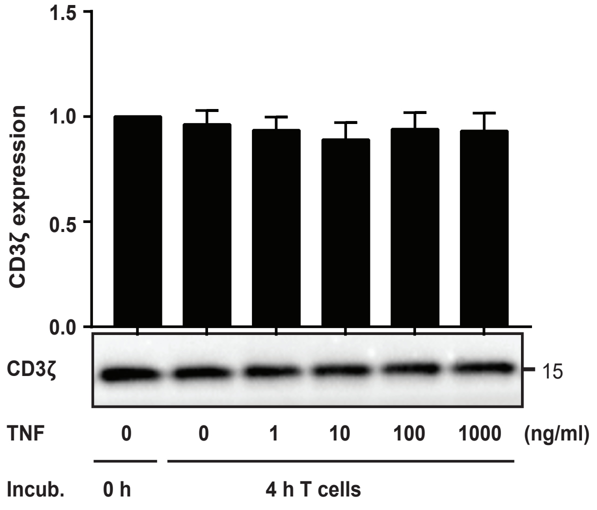

CD3ζ expression is not significantly affected by incubation and TNF treatment

Representative Western blot (lower panel) and quantification (upper panel) of CD3ζ from T cells lysed immediately after isolation (0 h, Procedure I, Fig. 1B) and after 4 hours of incubation (4 h T cells, Procedure I, Fig. 1B) with TNF (0 – 1000 ng/ml) as indicated. The Western blot is representative for Western blots obtained from 3 different biological experiments and the quantification shows the mean + SEM of the band densities of CD3ζ from Western blots obtained from these experiments. The positions of the relevant molecular weight marker and its molecular weight in kDa are given to the right of the Western blot.

Supplementary Figure 2
Levring et al.

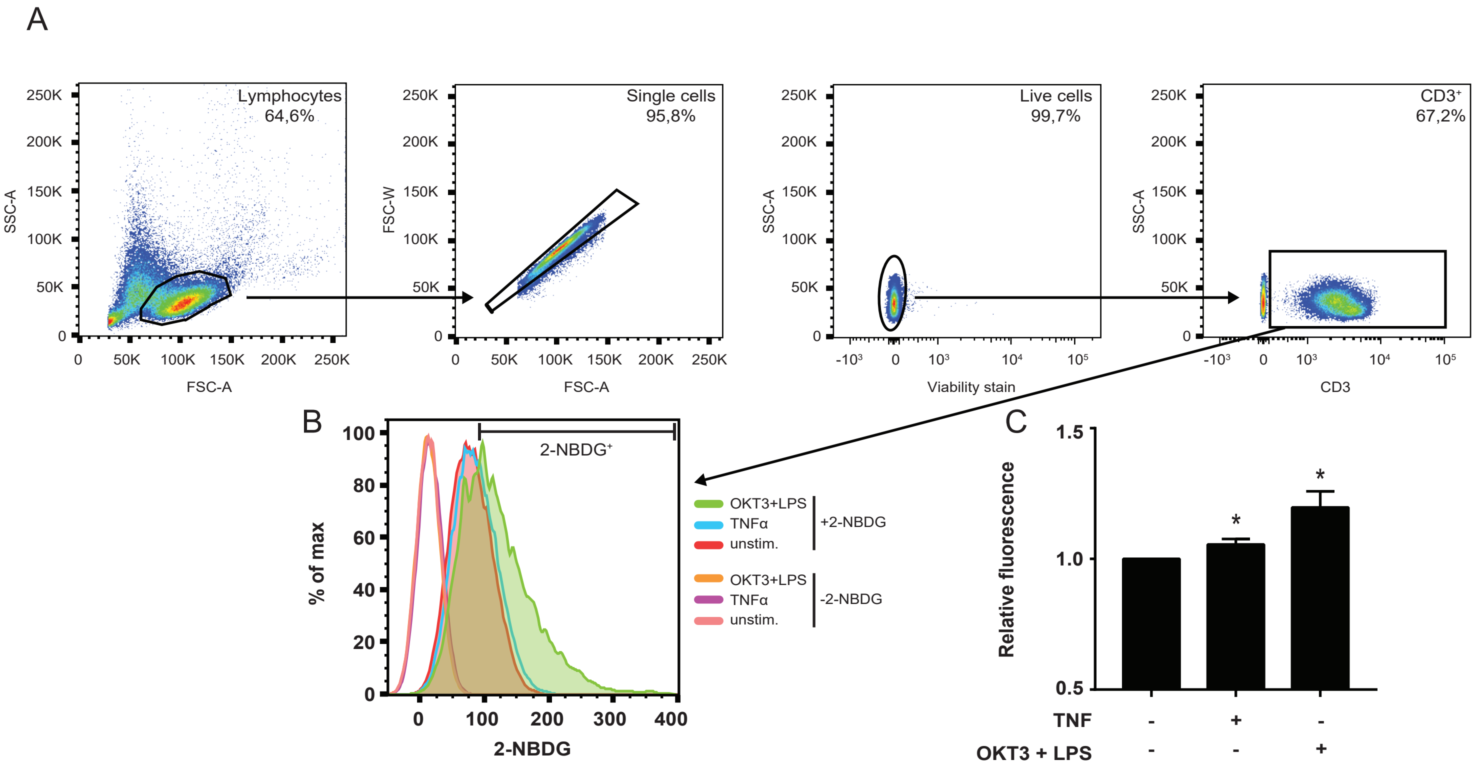

2-NBDG staining and gating strategy

Freshly drawn blood was diluted 1:1 in X-VIVO 15 medium and left unstimulated or stimulated with either TNF (10 ng/ml) or OKT3 (1000 ng/ml) and LPS (50 ng/ml) as indicated. The cells were incubated either in the presence or absence of 10 μM 2-NBDG. After 4 hours, the PBMC were isolated using Lymphoprep and subsequently stained for flow cytometric analysis. (A) Lymphocytes were gated based on the forward-scatter (FSC) side-scatter (SSC) dot plot. Subsequently singlet cells were isolated and next dead cells were gated out based on viability staining. (B) Lastly, $CD3^+$ T cells were gated and analyzed for 2-NBDG uptake with the positive gate set in relation to cells incubated in the absence of 2-NBDG. (C) Mean + SEM of the 2-NBDG mean fluorescence intensity of cells treated as indicated.

Supplementary Figure 3
Levring et al.

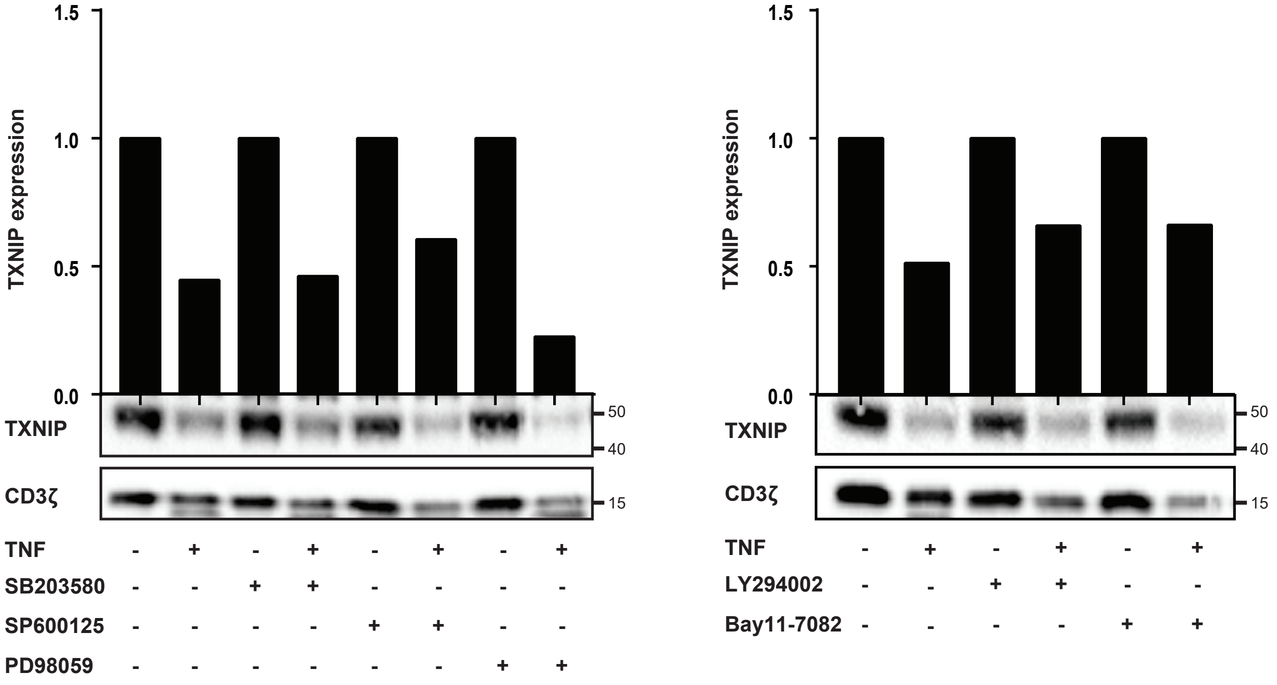

Inhibitors against p38, JNK, MEK, PI3K and IκB-α do not affect TNF-induced TXNIP down-regulation

Western blots (lower panels) and quantification (upper panels) of TXNIP with CD3ζ as loading control from T cells isolated from untreated blood and blood treated with TNF (10 ng/ml), p38 MAP kinase inhibitor SB203580 (10 μM), JNK inhibitor SP600125 (50 μM), MEK inhibitor PD98059 (20 μM), PI3K inhibitor LY294002 (5 μM) or IκB-α inhibitor Bay11-7082 (10 μM) for 4 hours as indicated. The positions of the relevant molecular weight markers and their molecular weight in kDa are given to the right of each western blot.
